# The Dignity of Work

**Published:** 1890-02-01

**Authors:** 


### Words of Consolation.

### THE DIGNITY OF WORK.

*(For Reading to the Sick.)*

The lives of many thousands of our fellow creatures are truthfully described in the following words, "They haste to rise up early, and so late take rest, and eat the bread of carefulness." And a very dull and anxious sort of existence it is in many cases where it is lived in one's own strength, for, except the Lord's blessing be upon our employments, our labour is but lost. Work has, like everything else, a right and a wrong point from which to view it, and the nearer we keep to the right one, the happier will our lives be. Work was given for man's happiness, since God put Adam into the garden of Eden to dress and to keep it, and it was not until after the fall that the curse fell on work and it became labour, when the ground was in future to bring forth thorns and thistles, and by the sweat of his face should man live. This is the point from which we are too apt to regard life. We look on our daily task as a burden, and consider every duty irksome which prevents our time being used for our own pleasure or profit. We envy those who appear to eat the bread of idleness, and this, with grumbling and discontent, fill up the measure of our days. But there is yet another side of the picture which it is our duty, and should be our pleasure, to contemplate. Labour has been dignified by our Lord's own example. During His youth and manhood, ere yet He came forth from obscurity to teach and save mankind, He dwelt at Nazareth, working with His supposed father, Joseph. In that lovely little village amidst the hills of Galilee, buried among the luxuriant foliage from which it takes its name, we may picture our Lord using "chisel, and saw, and plane," thus making noble for ever all handicrafts. We know it was so, for in after life the unbelieving Jews cast the reproach in His teeth, "Is not this the carpenter's son? Is not this the carpenter?" And they would not listen to His words. He, the great Artificer of the Universe, who made the world, and by the work of whose fingers the moon and stars were created, condescended to live like a common workman, and we are sure that not one single article was ever sent out imperfect. No scamped work, no hidden faults came from that workshop! Our Lord set us the example of doing all to God's glory, as in His earliest years He was anxious to be about His Father's business. Therefore our aim should be to give of our best, for then we shall be imitating our Great Example, and by putting our whole hearts and interest in our work, we are serving Him according to the old adage, "Labour is prayer." The maid who conscientiously sweeps all the corners of a room when the mistress's eye is not on her, and the youth who wastes neither his master's time nor material, are following in Christ's footsteps.

Think now, you who are lying on a sick bed, how far your work has been done properly! Has it been with repining and discontent, and so brought gloom on your home and ingratitude into your heart towards the God who made and saved you? Our Lord has taught us to pray daily, Thy will be done on earth (that is in the same spirit and as perfectly) as it is in heaven. There the angels are ever ready to do God's will, whether it be in small or great things. They fly as swiftly to take charge of Christ's little ones, or carry up the beggar into Abraham's bosom, as to protect a monarch or destroy a city. And if it please God to restore you to health, carry back to life the earnest resolve to do all to His glory, make His will your will, make His work your work.

It is not best in an inglorious ease
To sink and dull content.
Nay, best it is indeed
To spend ourselves upon the general good,
And, oft misunderstood,
To strive to lift the knees and limbs that bleed,
This is the best, the fullest meed.

Oh, labour thou art blest!

From all the earth, thy voice, a constant prayer,

Soars upward day and night:

A voice of aspiration after right;

A voice of effort yearning for its rest;

A voice of high hope conquering despair!

